# Examining healthcare needs and decisions to seek health services among Venezuelan migrants living in Trinidad and Tobago using Andersen’s Behavioral Model

**DOI:** 10.3389/fpubh.2023.1212825

**Published:** 2023-10-12

**Authors:** Nyla Lyons, Brendon Bhagwandeen

**Affiliations:** ^1^Medical Research Foundation of Trinidad and Tobago, Port of Spain, Trinidad and Tobago; ^2^School of Mathematical and Computer Sciences, Heriot-Watt University Malaysia, Putrajaya, Malaysia

**Keywords:** health service use, migrants, Trinidad and Tobago, Venezuelan, Andersen’s Behavioral Model

## Abstract

**Introduction:**

Beginning in 2016, Trinidad and Tobago experienced increasing flows of migrants and refugees from Venezuela. Through a Government Registration Exercise in 2019, followed by a Re-registration Exercise in 2020, migrants and refugees benefitted from access to publicly available primary care and emergency medical services. By applying Andersen’s Behavioral Model for Health Service Use, our study examined the non-communicable disease care needs of migrants, and factors influencing their decision to seek public and private health services.

**Method:**

Between September and December 2020, a health questionnaire was administered via telephone to *n* = 250 migrants from Venezuela. Descriptive statistics summarized the constructs of Andersen’s Behavioral Model. The model comprised of predisposing factors including migrants’ social characteristics; enabling factors namely monthly earnings, education level and most trusted source of information on medical needs; need for care factors such as migrants self-reported health status, presence of non-communicable health conditions and having visited a doctor in the past 12 months; and the outcome variables which were migrants’ decisions to seek public and private health services. Pearson *χ*^2^ tests, odds ratios and multivariable logistic regression with backward elimination examined the factors influencing a migrant’s decision to seek health services.

**Results:**

Overall, 66.8% of migrants reported they would seek public health services, while 22.4% indicated they would seek private health services. Predisposing factors namely length of time residing in Trinidad and Tobago (*p* = 0.031) and living with family/friends (*p* = 0.049); the enabling factor of receiving information from publicly available sources (*p* = 0.037); and the need for care factor of visiting a doctor for a physical health problem (*p* = 0.010) were significant correlates of their decision to seek care in the public sector. Predisposing factors namely living with family/friends (*p* = 0.020) and the enabling factor of having difficulty accessing healthcare services (*p* = 0.045) were significant correlates of their decision to seek care from private providers.

**Discussion:**

Our findings demonstrated the positive association between social networks and a migrant’s decision to use public and private health services, thus underscoring the importance of family and friends in facilitating health service use, promoting proper health practices and preventing diseases. Overall, the use of Andersen’s Behavioral Model aided in identifying the factors associated with the use of health services by Venezuelan migrants in Trinidad and Tobago. However, further studies are needed to better understand their need for ongoing care, to inform policy, and to plan targeted health interventions for addressing the gaps in health service access, barriers and use.

## 1. Introduction

As of August 2020, the United Nations High Commissioner for Refugees (UNHCR) estimated that 5.4 million Venezuelan refugees and migrants fled their country in search of food, shelter, and healthcare because of ongoing political and economic instability in Venezuela. Up to 85% of Venezuelan refugees and migrants resided in Latin American countries such as Colombia (1.7 million), followed by Peru (870,000), Ecuador (385,000), and Chile (371,000). Many were also seeking safety in the Caribbean, including the countries of Aruba, Curacao, the Dominican Republic, Guyana, and Trinidad and Tobago (1).

Beginning in 2016, Trinidad and Tobago experienced increasing flows of refugees and migrants from Venezuela. By the end of 2017, the UNHCR reported a total of 2,700 refugees seeking asylum and by April 2018, the number of reported refugees increased to over 5,300. By the end of 2019, the UNHCR estimated just over 21,000 refugees and migrants from Venezuela residing in Trinidad and Tobago. This number was expected to increase to 33,400 by the end of 2020 (2). A report by Refugees International in 2019 put the number of Venezuelans on the islands of Trinidad and Tobago at 40,000, giving the highest *per capita* Venezuelan population in the English-speaking Caribbean (2).

Non-communicable diseases (NCDs) represent a significant share of mortality and morbidity among persons residing in Venezuela. In 2021, it was estimated that 18.8 million Venezuelans worldwide lacked access to health services, including 10.4 million who were diagnosed with chronic diseases (3). Conditions surrounding the migration process may increase exposure and vulnerability to NCD risk factors. Upon arrival in host countries, it has been shown that migrants were further exposed to lifestyle risk factors and behavioral changes which increased their risk and vulnerability. Therefore, these circumstances left migrants at a higher risk of developing or worsening existing health conditions (3). Migrants’ health might be further compromised because of their healthcare experiences such as language and/or cultural differences, cost of medications, discrimination, xenophobia, and lack of information on where to access health services, thus affecting the continuity of treatment which is critical for many NCDs.

In Trinidad and Tobago, persons in need of international protection remained subject to the provisions of the 1976 Immigration Act. The Government acceded to the 1951 Geneva Convention on the Status of Refugees, and its 1967 Protocol in 2014 (4). In 2020, a draft policy was developed to address the provision of public healthcare services to non-nationals (5), and as of 2023, it is yet to be officially implemented. This intensified challenges for migrants and refugees who already faced barriers getting the care they needed. As a result of public health measures, including the closure of non-essential services and businesses during the most recent pandemic, this was exacerbated and it was reported that Venezuelan refugees and migrants in Trinidad and Tobago were greatly impacted by a loss of income (6), further compromising their access to health services. Currently, there has been no national policy in Trinidad and Tobago consistent with international law and standards governing migrants’ access to ongoing healthcare services. In June 2019, in response to the increasing number of refugees and migrants from Venezuela, the Government of the Republic of Trinidad and Tobago conducted a Registration Exercise whereby all refugees and migrants (including those residing illegally) were given an opportunity to obtain “one-year legal status” in the country. Successful registrants were granted one-year work permits and benefitted from access to publicly available emergency medical and primary care services. However, access to secondary and tertiary healthcare remained limited. This was documented in 2020 in the policy for treating with non-nationals for the provision of public health services. Access to ongoing medical care and specialized health services such as the treatment of cancer and for surgeries were not covered in the policy. It was further advised that migrants seeking ongoing medical services and specialized care may do so at their own expense. In March 2020, a Re-registration Exercise was conducted for migrants previously registered in 2019, providing them with a six-month extension (5).

The burden of non-communicable diseases among migrants in Trinidad and Tobago is unclear and moreover, their health needs relating to NCDs are not well understood. The lack of legal documents can prove to be a major barrier to accessing care at public facilities as patients are asked to produce these documents at the registration counter. Workers at the healthcare facilities are required to inform the authorities of undocumented migrants. Despite this, many healthcare workers in public health centers still provided the required care to migrants. On the other hand, private facilities would generally treat patients regardless of their immigration status. However, in both the public and private setting, language and communication were major barriers faced. Therefore, by applying Andersen’s Behavioral Model for Health Service Use, this study examined the medical needs of Venezuelan migrants residing in Trinidad and Tobago and the associations between predisposing, enabling and needs factors with their decisions to seek public and private health services. With the advent of public health emergencies such as in the case of the COVID-19 pandemic, health inequalities were exacerbated for refugees and asylum seekers because economic and social hardships left them at risk of worsening health conditions. In addition, the capacities of health systems may be stretched, further compounding issues related to access and use of public health services for refugees and migrant subpopulations. Therefore, this study also had implications for development of a comprehensive national policy prioritizing refuges and integrating the health of refugees and migrants as part of the national response during public health emergencies.

## 2. Materials and methods

### 2.1. Analytical framework

**Figure 1:**
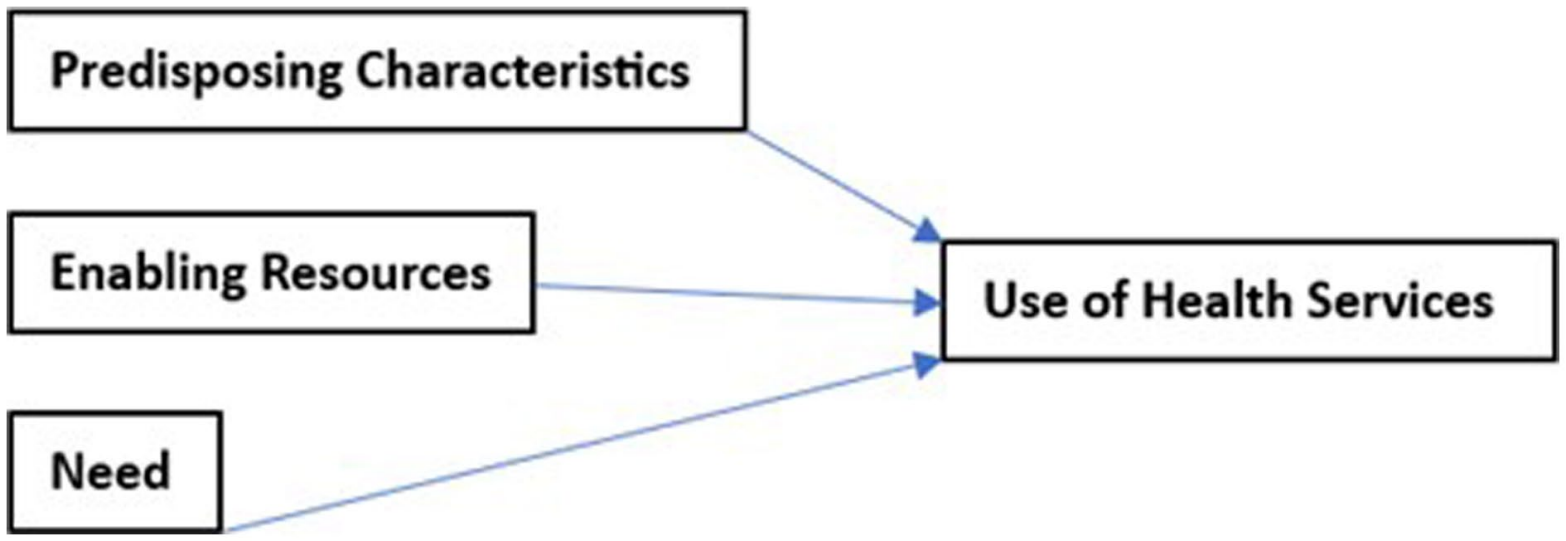
General Framework of Andersen’s Behavioral Model.

Andersen’s Behavioral Model for Health Service Use has been applied in several studies in a variety of settings and across populations to assess health seeking behaviors. The model is a well-validated theoretical framework aimed at understanding determinants of health service utilization, taking into account both individual and social determinants (7). The model asserts that access to and use of health services are influenced by three main categories of factors: (1) predisposing factors (social characteristics), (2) enabling factors (resource availability), and (3) need factors (quest for solutions to illness) (8) (Figure 1).

### 2.2. Study context

The Medical Research Foundation of Trinidad and Tobago (MRFTT) was established in March 1997 as a non-governmental organization (NGO) to investigate the epidemiology of HIV and HTLV-1. It is the largest HIV Treatment and Care Centre in Trinidad and Tobago. In 2002, with the increasing prevalence of HIV/AIDS, the Government of the Republic of Trinidad and Tobago commissioned the MRFTT to deliver prevention programs and treatment to persons diagnosed with HIV. Beginning in 2018, the MRFTT expanded the programs to target migrant subpopulations with non-communicable disease-risk prevention, treatment and management through health outreach activities. Given the inflows of Venezuelan migrants, it was important to examine the health needs and use of health services by this minority subpopulation and understand the extent of diseases and uptake of health services which could assist in reducing new and/or worsening health conditions.

### 2.3. Study design and participants

This cross-sectional study was carried out from September to December 2020 among Venezuelan migrants residing in Trinidad and Tobago via telephone interviews using a structured questionnaire due to the COVID-19 pandemic restrictions. Each interview was done by one of two trained bilingual nurses. The study was facilitated by the MRFTT.

The study used two sampling strategies since the subpopulation of Venezuelan migrants in Trinidad and Tobago was largely hidden. A convenience sample of migrants was reached from persons responding to flyers with information about the study, that were distributed to a large community-based organization providing refugee support services to Venezuelan migrants. These migrants would then share details of the study for snowball sampling to be used in recruiting further participants. Responses came from a sample of *n* = 250 Venezuelan migrants residing in Trinidad and Tobago. Persons were given the incentive of free health check-ups for participating.

The eligibility criteria included persons of Venezuelan nationality living in Trinidad and Tobago between 3 months and 5 years. Persons meeting the criteria were briefed about the study protocol and were asked to provide their consent to participate. Interviews were conducted in Spanish by the nurses, responses were translated and then recorded in English to a Microsoft Excel spreadsheet stored on a password protected computer. Only members of the research team had access to the data file.

### 2.4. Study questionnaire and data collection

**Figure 2:**
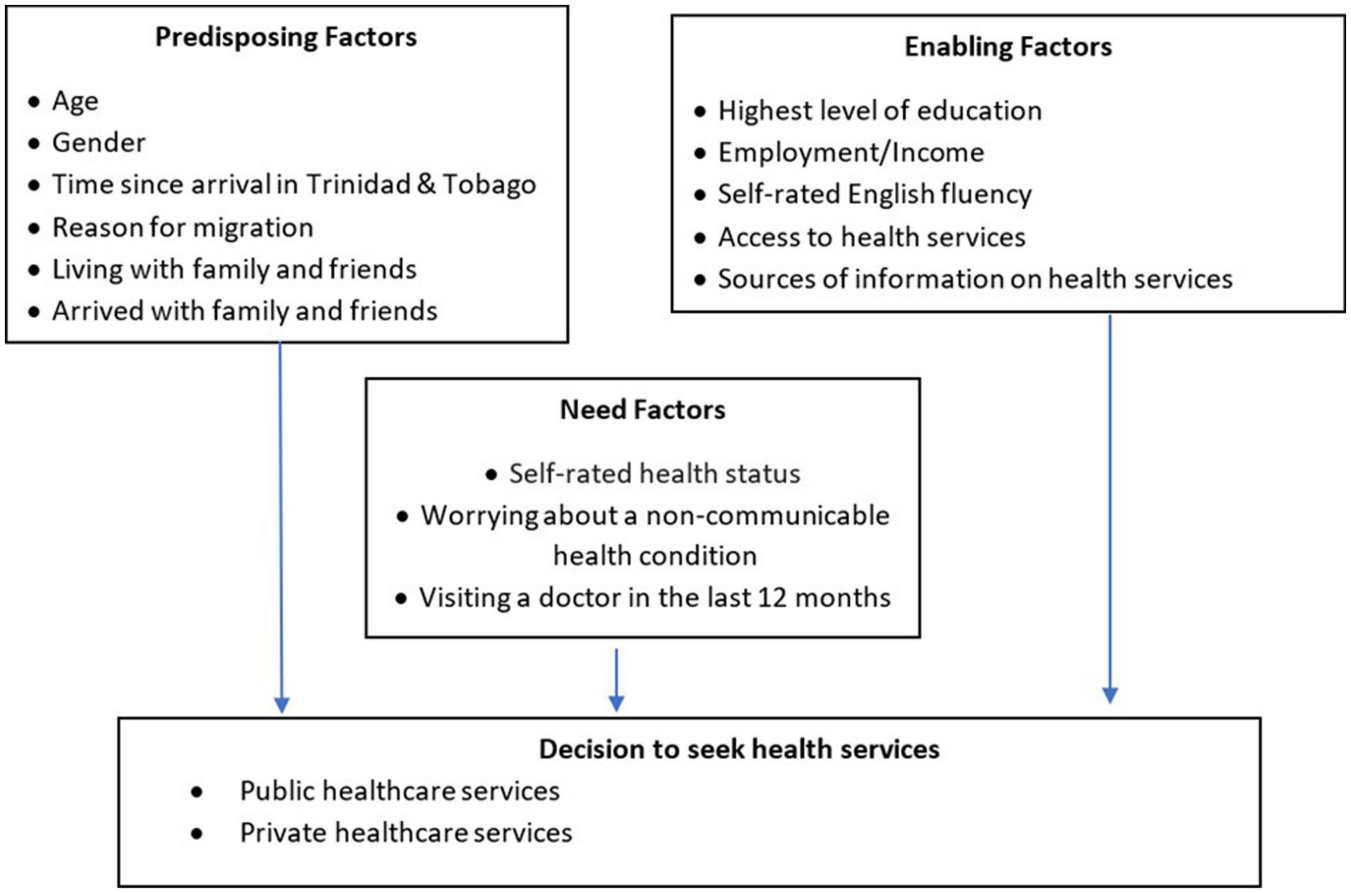
Andersen’s Behavioral Model for Health Service Use in this study.

Guided by Andersen’s Behavioral Model for Health Service Use, predisposing factors in our study were social characteristics including age, gender, time since arrival in Trinidad and Tobago, main reason for migration, living alone or with family/friends and having arrived in Trinidad and Tobago alone or with family/friends. Enabling factors explained barriers and facilitators to health service use such as education level, income earned, self-rated English fluency, knowledge of where to access healthcare, having difficulties accessing care and most trusted source of information about medical needs. Need factors were overall self-rated health status, worrying about a diagnosed non-communicable health condition (mental or physical) and having visited a doctor in the past 12 months for a diagnosed non-communicable health condition (mental or physical) as shown in Figure 2. A questionnaire using these factors was developed and translated into Spanish for interviews.

The outcome variables in this study were migrants’ decisions to seek public health services and private health services. Public health services included public hospitals, community health centers and emergency medical services. Private health services included private doctors, private hospitals, and pharmacists.

The questionnaire was pre-tested using a convenience sample of migrants selected from persons with similar characteristics to those that were intended to participate in the study. This helped in identifying potential problems in the language, structure and design of the questionnaire. Some minor adjustments were subsequently made to better facilitate the flow of the interview process. Each interview took between 10 and 15 min to complete.

### 2.5. Statistical analysis

Descriptive statistics for categorical variables were presented as frequencies with percentages and were used to characterize baseline distributions of study variables. Univariate associations between predisposing, enabling and need factors with a migrant’s decision to use public and private health services were observed using Pearson *χ*^2^ tests or Fisher’s exact tests where appropriate and unadjusted odds ratios (OR) with 95% confidence intervals (CI). Baseline differences were also compared using Pearson *χ*^2^ tests or Fisher’s exact tests. Predisposing, enabling and need factors based on Andersen’s Behavioral Model were used in multivariable logistic regression analyses to observe significant correlates and adjusted odds ratios (AOR) for migrants deciding to seek public and private health services in Trinidad and Tobago. Backward elimination was used to select the correlates which best explained their decisions. Statistical significance was set at *p* < 0.05.

### 2.6. Ethical approval

Permission to conduct this study was granted by the University of the West Indies (UWI) Institutional Review Board (IRB) in September 2020.

## 3. Results

### 3.1. Social characteristics and health needs of migrants

Table 1 summarized the social characteristics of the migrants in this study. A total of *n* = 250 Venezuelan migrants residing in Trinidad and Tobago were recruited. Most participants were under 40 years of age (*n* = 165, 66%), female (*n* = 149, 59.6%) and have been residing in Trinidad and Tobago for at least 12 months (*n* = 213, 85.2%). Approximately 90% of the migrants in this study left Venezuela because of economic or political reasons and now live in Trinidad and Tobago with either family or friends. Most of the participants completed formal schooling (*n* = 221, 88.4%), were earning under $1,000 TT each month (*n* = 145, 58%) and self-reported low levels of English fluency (*n* = 157, 62.8%). Chaguanas/Couva (*n* = 39, 15.6%), San Fernando (*n* = 47, 18.8%) and Point Fortin (*n* = 33, 13.2%) were the common areas of residence for these migrants.

**Table 1:** Social characteristics of migrants.

Variable	*n*	%
Age		
20–29 years	72	28.8
30–39 years	93	37.2
40–49 years	59	23.6
≥50 years	26	10.4
Total	250	100.0
Gender		
Male	101	40.4
Female	149	59.6
Total	250	100.0
Time since migrating to Trinidad and Tobago		
<12 months	37	14.8
≥12 months	213	85.2
Total	250	100.0
Main reason for migration		
Economical/Political	227	90.8
Asylum seeker/Escape violence	11	4.4
Family reunification	12	4.8
Total	250	100.0
Household dynamic		
Lives alone	22	8.8
Lives with family/friends	228	91.2
Total	250	100.0
Education level		
No formal education	29	11.6
Elementary schooling	94	37.6
High school	120	48.0
College/University schooling	7	2.8
Total	250	100.0
Monthly earnings		
<$1,000 TT	145	58.0
$1,000–$3,000 TT	70	28.0
>$3,000 TT	35	14.0
Total	250	100.0
English fluency		
Low	157	62.8
Moderate	81	32.4
High	12	4.8
Total	250	100.0
Approximate area of residence in Trinidad and Tobago		
Arima/Tunapuna/Piarco	15	6.0
Chaguanas/Couva	39	15.6
Mayaro/Rio Claro	23	9.2
Penal/Debe/Siparia	18	7.2
Point Fortin	33	13.2
Port of Spain/San Juan/Laventille	14	5.6
Princess Town	16	6.4
San Fernando	47	18.8
Other	45	18.0
Total	250	100.0

Table 2 summarized the health status and health needs of these migrants. Most of the participants believed that they were in “good health” (*n* = 152, 60.8%). Approximately 30% of the participants reported diagnosed mental and physical non-communicable health problems which worried them, with 53 (21.2%) believing that they needed to visit a doctor for their mental health problems, while 113 (45.2%) believed that they needed to visit a physician or health center for their physical health problems. Twenty-nine of the participants in this study (11.6%) had some knowledge of where to access healthcare in Trinidad and Tobago upon arrival, with 78 (31.2%) experiencing difficulties in accessing healthcare since residing in the country. Approximately two-thirds of the migrants (*n* = 167, 66.8%) reported that official healthcare providers were their most trusted sources for information regarding their medical needs. Overall, 167 migrants (66.8%) indicated that they would seek public health services, while 56 (22.4%) would seek private healthcare in Trinidad and Tobago.

**Table 2:** Health status and health needs of migrants.

Variable	*n*	%
View of health (self-rated)		
Poor health	24	9.6
Fair health	74	29.6
Good health	152	60.8
Total	250	100.0
Diagnosed mental health problem		
No	171	68.4
Yes	79	31.6
Total	250	100.0
Diagnosed physical health problem		
No	180	72.0
Yes	70	28.0
Total	250	100.0
Needed to visit doctor for mental health problem		
No	197	78.8
Yes	53	21.2
Total	250	100.0
Needed to visit doctor for physical health problem		
No	137	54.8
Yes	113	45.2
Total	250	100.0
Knowledge of where to access healthcare in Trinidad and Tobago upon arrival		
No	221	88.4
Yes	29	11.6
Total	250	100.0
Had difficulties accessing healthcare since in Trinidad and Tobago		
No	172	68.8
Yes	78	31.2
Total	250	100.0
Most trusted source of information on medical needs		
Official healthcare providers	167	66.8
Social networks	20	8.0
Internet	49	19.6
Other	14	5.6
Total	250	100.0
Decision to seek public health services		
No	83	33.2
Yes	167	66.8
Total	250	100.0
Decision to seek private health services		
No	194	77.6
Yes	56	22.4
Total	250	100.0
Decision to seek health services		
No	48	19.2
Yes	202	80.8
Total	250	100.0

### 3.2. Differences in migrants’ decisions to seek public and private health services according to social characteristics

Table 3 summarized the differences in migrants’ decisions to seek public and private health services according to their social characteristics.

**Table 3:** Differences in migrants’ decision to seek public and private health services according to social characteristics, row %.

Variable	Decision to seek public healthcare			Decision to seek private healthcare		
	No	Yes	*p*-value	No	Yes	*p*-value
Age			0.604			0.902
20–29 years	27 (37.5)	45 (62.5)		56 (77.8)	16 (22.2)	
30–39 years	30 (32.3)	63 (67.7)		74 (79.6)	19 (20.4)	
40–49 years	20 (33.9)	39 (66.1)		45 (76.3)	14 (23.7)	
≥50 years	6 (23.1)	20 (76.9)		19 (73.1)	7 (26.9)	
Gender			0.688			0.297
Male	35 (34.7)	66 (65.3)		75 (74.3)	26 (25.7)	
Female	48 (32.2)	101 (67.8)		119 (79.9)	30 (20.1)	
Time since migrating to Trinidad and Tobago			0.031			0.247
<12 months	18 (48.6)	19 (51.4)		26 (70.3)	11 (29.7)	
≥12 months	65 (30.5)	148 (69.5)		168 (78.9)	45 (21.1)	
Main reason for migration			0.007			0.570
Economical/Political	77 (33.9)	150 (66.1)		174 (76.7)	53 (23.3)	
Asylum seeker/Escape violence	6 (54.5)	5 (45.5)		9 (81.8)	2 (18.2)	
Family reunification	–	12 (100.0)		11 (91.7)	1 (8.3)	
Household dynamic			0.007			0.029
Lives alone	13 (59.1)	9 (40.9)		13 (59.1)	9 (40.9)	
Lives with family/friends	70 (30.7)	158 (69.3)		181 (79.4)	47 (20.6)	
Education level			0.194			0.266
No formal education	13 (44.8)	16 (55.2)		24 (82.8)	5 (17.2)	
Elementary schooling	35 (37.2)	59 (62.8)		78 (83.0)	16 (17.0)	
High school	34 (28.3)	86 (71.7)		87 (72.5)	33 (27.5)	
College/University schooling	1 (14.3)	6 (85.7)		5 (71.4)	2 (28.6)	
Monthly earnings			0.032			0.311
<$1,000 TT	57 (39.3)	88 (60.7)		117 (80.7)	28 (19.3)	
$1,000–$3,000 TT	15 (21.4)	55 (78.6)		50 (71.4)	20 (28.6)	
>$3,000 TT	11 (31.4)	24 (68.6)		27 (77.1)	8 (22.9)	
English fluency			0.737			0.770
Low	53 (33.8)	104 (66.2)		123 (78.3)	34 (21.7)	
Moderate	25 (30.9)	56 (69.1)		61 (75.3)	20 (24.7)	
High	5 (41.7)	7 (58.3)		10 (83.3)	2 (16.7)	
Approximate area of residence in Trinidad and Tobago			0.885			0.597
Arima/Tunapuna/Piarco	6 (40.0)	9 (60.0)		12 (80.0)	3 (20.0)	
Chaguanas/Couva	11 (28.2)	28 (71.8)		26 (66.7)	13 (33.3)	
Mayaro/Rio Claro	9 (39.1)	14 (60.9)		17 (73.9)	6 (26.1)	
Penal/Debe/Siparia	4 (22.2)	14 (77.8)		13 (72.2)	5 (27.8)	
Point Fortin	13 (39.4)	20 (60.6)		25 (75.8)	8 (24.2)	
Port of Spain/San Juan/Laventille	6 (42.9)	8 (57.1)		12 (85.7)	2 (14.3)	
Princess Town	5 (31.3)	11 (68.8)		13 (81.3)	3 (18.8)	
San Fernando	16 (34.0)	31 (66.0)		41 (87.2)	6 (12.8)	
Other	13 (28.9)	32 (71.1)		35 (77.8)	10 (22.2)	

Significant differences in a migrant’s decision to seek public health services were observed when the length of time they resided in Trinidad and Tobago (*p* = 0.031), primary reason for migrating (*p* = 0.007), household dynamic (*p* = 0.007), and monthly earnings (*p* = 0.032) were considered. There was a significantly higher proportion of migrants residing in Trinidad and Tobago for at least 12 months (69.5%) who would seek public health services compared to those living in Trinidad and Tobago for less than 12 months (51.4%). All persons who migrated to reunite with their family would seek public health services; this was followed by persons who migrated for economic or political reasons (66.1%) and asylum seekers (45.5%). There was a significantly higher proportion of migrants living with family or friends (69.3%) who would seek public health services compared to those who lived alone (40.9%). Migrants earning $1,000–$3,000 TT each month (78.6%) had the highest proportion of persons who would seek public health services, followed by those who earned more than $3,000 TT (68.6%) and then less than $1,000 TT each month (60.7%). However, significant differences in migrant’s decision to seek public health services were not observed when age (*p* = 0.604), gender (*p* = 0.688), highest level of education obtained (*p* = 0.194), English fluency (*p* = 0.737) and approximate area of residence in Trinidad and Tobago (*p* = 0.885) were considered.

A significant difference in migrants’ decisions to seek private health services was observed when their household dynamic (*p* = 0.029) was considered. There was a significantly lower proportion of migrants living with family or friends (20.6%) who would seek private health services compared to those who lived alone (40.9%). Significant differences in a migrant’s decision to seek private health services were not observed when categories of age (*p* = 0.902), gender (*p* = 0.297), length of time residing in Trinidad and Tobago (*p* = 0.247), primary reason for migrating (*p* = 0.570), highest level of education obtained (*p* = 0.266), monthly earnings (*p* = 0.311), English fluency (*p* = 0.770), and approximate area of residence in Trinidad and Tobago (*p* = 0.597) were considered.

### 3.3. Associations between migrants’ decisions to seek public and private health services with social characteristics

Table 4 provided the univariate associations and unadjusted odds ratios with 95% confidence intervals of a migrant’s decision to seek public and private health services with their social characteristics.

**Table 4:** Univariate associations and unadjusted odds ratios (with 95% CI) of migrants’ decisions to seek public and private health services with social characteristics.

Variable	Decision to seek public healthcare (Ref: Decided not to seek public healthcare)			Decision to seek private healthcare (Ref: Decided not to seek private healthcare)		
	Unadjusted OR	95% CI	*p*-value	Unadjusted OR	95% CI	*p*-value
Age			0.610			0.903
20–29 years	Ref	Ref	–	Ref	Ref	–
30–39 years	1.260	0.661–2.403	0.483	0.899	0.424–1.903	0.899
40–49 years	1.170	0.570–2.403	0.669	1.089	0.481–2.466	0.838
≥50 years	2.000	0.714–5.600	0.187	1.289	0.461–3.610	0.628
Gender			0.688			0.298
Male	Ref	Ref	–	Ref	Ref	–
Female	1.116	0.654–1.905	0.688	0.727	0.399–1.324	0.298
Time since migrating to Trinidad and Tobago			0.033			0.249
<12 months	Ref	Ref	–	Ref	Ref	–
≥12 months	2.157	1.063–4.377	0.033	0.633	0.291–1.378	0.249
Main reason for migration			0.393			0.486
Economical/Political	Ref	Ref	–	Ref	Ref	–
Asylum seeker/Escape violence	0.428	0.127–1.446	0.172	0.730	0.153–3.481	0.692
Family reunification	–	–	–	0.298	0.038–2.365	0.252
Household dynamic			0.010			0.034
Lives alone	Ref	Ref	–	Ref	Ref	–
Lives with family/friends	3.260	1.332–7.981	0.010	0.375	0.151–0.930	0.034
Education level			0.195			0.273
No formal education	Ref	Ref	–	Ref	Ref	–
Elementary schooling	1.370	0.590–3.182	0.465	0.985	0.327–2.968	0.978
High school	2.055	0.894–4.725	0.090	1.821	0.641–5.169	0.260
College/University schooling	4.875	0.519–45.789	0.166	1.920	0.287–12.862	0.501
Monthly earnings			0.035			0.315
<$1,000 TT	Ref	Ref	–	Ref	Ref	–
$1,000–$3,000 TT	2.375	1.226–4.600	0.010	1.671	0.862–3.242	0.129
>$3,000 TT	1.413	0.643–3.106	0.389	1.238	0.508–3.016	0.638
English fluency			0.739			0.772
Low	Ref	Ref	–	Ref	Ref	–
Moderate	1.142	0.642	2.031	1.186	0.631–2.231	0.597
High	0.713	0.216–2.355	0.580	0.724	0.151–3.460	0.685
Approximate area of residence in Trinidad and Tobago			0.890			0.627
Arima/Tunapuna/Piarco	Ref	Ref	–	Ref	Ref	–
Chaguanas/Couva	1.697	0.488–5.902	0.406	2.000	0.479–8.354	0.342
Mayaro/Rio Claro	1.037	0.274–3.920	0.957	1.412	0.294–6.790	0.667
Penal/Debe/Siparia	2.333	0.512–10.638	0.274	1.538	0.301–7.870	0.605
Point Fortin	1.026	0.295–3.569	0.968	1.280	0.287–5.707	0.746
Port of Spain/San Juan/Laventille	0.899	0.203–3.901	0.876	0.667	0.094–4.733	0.685
Princess Town	1.467	0.335–6.430	0.612	0.923	0.155–5.486	0.930
San Fernando	1.292	0.390–4.273	0.675	0.585	0.127–2.698	0.492
Other	1.641	0.486–5.545	0.425	1.143	0.269–4.859	0.856

Ref, Reference category.

The length of time migrants resided in Trinidad and Tobago was significantly associated with their decision to seek public health services in the country (*p* = 0.033). The odds of migrants living in Trinidad and Tobago for more than 12 months seeking public health services were over twice the odds of migrants residing in the country for less than 12 months (OR 2.157, 95% CI 1.063–4.377). The household dynamic of participants in this study was another independent factor that was significantly associated with their decision to seek public health services in Trinidad and Tobago (*p* = 0.010). The odds of persons living with family or friends seeking public health services were over three times the odds of persons living alone (OR 3.260, 95% CI 1.332–7.981). Monthly earnings were also significantly associated with a migrant’s decision to seek public health services (*p* = 0.035). Persons earning between $1,000–$3,000 TT each month had greater odds of seeking public health services relative to migrants earning less than $1,000 TT per month (OR 2.375, 95% CI 1.226–4.600). Migrants’ age, gender, primary reason for migrating, highest level of education obtained, English fluency and approximate area of residence were not observed to be statistically significantly associated with their decision to seek public health services in Trinidad and Tobago (*p* > 0.05).

The household dynamic of migrants was an independent factor that was significantly associated with their decision to seek private health services in Trinidad and Tobago (*p* = 0.034). The odds of persons living with family or friends seeking private health services were lower than the odds of persons living alone (OR 0.375, 95% CI 0.151–0.930). Other social characteristics of migrants were not statistically significantly associated with their decisions to seek private health services in Trinidad and Tobago (*p* > 0.05).

### 3.4. Differences in migrants’ decisions to seek public and private health services according to health status and health needs

Table 5 summarized the differences in migrants’ decisions to seek public and private health services according to their health status and health needs.

**Table 5:** Differences in migrants’ decision to access public and private health services according to health status and health needs, row %.

Variable	Decision to seek public healthcare			Decision to seek private healthcare		
	No	Yes	*p*-value	No	Yes	*p*-value
View of health (self-rated)			0.558			0.458
Poor health	9 (37.5)	15 (62.5)		20 (83.3)	4 (16.7)	
Fair health	21 (28.4)	53 (71.6)		60 (81.1)	14 (18.9)	
Good health	53 (34.9)	99 (65.1)		114 (75.0)	38 (25.0)	
Diagnosed mental health problem			0.824			0.921
No	56 (32.7)	115 (67.3)		133 (77.8)	38 (22.2)	
Yes	27 (34.2)	52 (65.8)		61 (77.2)	18 (22.8)	
Diagnosed physical health problem			0.943			0.818
No	60 (33.3)	120 (66.7)		139 (77.2)	41 (22.8)	
Yes	23 (32.9)	47 (67.1)		55 (78.6)	15 (21.4)	
Needed to visit doctor for mental health problem			0.645			0.286
No	64 (32.5)	133 (67.5)		150 (76.1)	47 (23.9)	
Yes	19 (35.8)	34 (64.2)		44 (83.0)	9 (17.0)	
Needed to visit doctor for physical health problem			0.005			0.008
No	56 (40.9)	81 (59.1)		115 (83.9)	22 (16.1)	
Yes	27 (23.9)	86 (76.1)		79 (69.9)	34 (30.1)	
Knowledge of where to access healthcare in Trinidad and Tobago upon arrival			0.792			0.814
No	74 (33.5)	147 (66.5)		171 (77.4)	50 (22.6)	
Yes	9 (31.0)	20 (69.0)		23 (79.3)	6 (20.7)	
Had difficulties accessing healthcare since in Trinidad and Tobago			0.259			0.034
No	61 (35.5)	111 (64.5)		127 (73.8)	45 (26.2)	
Yes	22 (28.2)	56 (71.8)		67 (85.9)	11 (14.1)	
Most trusted source of information on medical needs			0.012			0.129
Official healthcare providers	44 (26.3)	123 (73.7)		125 (74.9)	42 (25.1)	
Social networks	9 (45.0)	11 (55.0)		17 (85.0)	3 (15.0)	
Internet	24 (49.0)	25 (51.0)		38 (77.6)	11 (22.4)	
Other	6 (42.9)	8 (57.1)		14 (100.0)	–	

Significant differences in a migrant’s decision to seek public health services were observed when their need to visit a doctor for a physical health problem (*p* = 0.005) and their main source of information relating to medical needs (*p* = 0.012) were considered. A significantly higher proportion of migrants who needed to visit a doctor for a physical health problem (76.1%) would seek public health services compared to persons who did not need to visit a doctor for a physical health problem (59.1%). The proportion of migrants who trusted official healthcare providers for information concerning their medical needs (73.7%) who would seek public health services was significantly higher than persons who trusted social networks (55%), internet sources (51%) or an alternative source (57.1%). Significant differences in a migrant’s decision to seek public health services were not observed when their self-rated health status (*p* = 0.558), having a diagnosed mental health problem (*p* = 0.824), having a diagnosed physical health problem (*p* = 0.943), needing to visit a doctor for a mental health problem (*p* = 0.645), knowing where to access healthcare in Trinidad and Tobago upon arrival (*p* = 0.792) and having difficulties accessing healthcare since residing in Trinidad and Tobago (*p* = 0.259) were considered.

Significant differences in a migrant’s decision to seek private health services were observed when their need to visit a doctor for a physical health problem (*p* = 0.008) and having difficulties accessing healthcare since residing in Trinidad and Tobago (*p* = 0.034) were considered. A significantly higher proportion of migrants who needed to visit a doctor for a physical health problem (30.1%) would seek private health services compared to persons who did not need to visit a doctor for a physical health problem (16.1%), while a significantly lower proportion of migrants who experienced difficulties accessing healthcare since residing in Trinidad and Tobago (14.1%) would seek private health services compared to persons who did not experience any difficulties accessing health services (26.2%). Significant differences in a migrant’s decision to seek private health services were not observed when their self-rated health status (*p* = 0.458), having a diagnosed mental health problem (*p* = 0.921), having a diagnosed physical health problem (*p* = 0.818), needing to visit a doctor for a mental health problem (*p* = 0.286), knowing where to access healthcare in Trinidad and Tobago upon arrival (*p* = 0.814) and their most trusted source of information relating to medical needs (*p* = 0.129) were considered.

### 3.5. Associations between migrants’ decisions to seek public and private health services with health status and health needs

Table 6 provided the univariate associations and unadjusted odds ratios with 95% confidence intervals of migrants’ decisions to seek public and private health services with their health status and health needs.

**Table 6:** Univariate associations and unadjusted odds ratios (with 95% CI) of migrants’ decision to access healthcare services with health status/needs.

Variable	Decision to seek public healthcare (Ref: Decided not to seek public healthcare)			Decision to seek private healthcare (Ref: Decided not to seek private healthcare)		
	Unadjusted OR	95% CI	*p*-value	Unadjusted OR	95% CI	*p*-value
View of health (self-rated)			0.559			0.461
Poor health	Ref	Ref	–	Ref	Ref	–
Fair health	1.514	0.575–3.989	0.401	1.167	0.344–3.956	0.805
Good health	1.121	0.460–2.732	0.802	1.667	0.536–5.183	0.378
Diagnosed mental health problem			0.824			0.921
No	Ref	Ref	–	Ref	Ref	–
Yes	0.938	0.534–1.649	0.824	1.033	0.546–1.954	0.921
Diagnosed physical health problem			0.943			0.818
No	Ref	Ref	–	Ref	Ref	–
Yes	1.022	0.568–1.838	0.943	0.925	0.474–1.805	0.818
Needed to visit doctor for mental health problem			0.645			0.289
No	Ref	Ref	–	Ref	Ref	–
Yes	0.861	0.456–1.626	0.645	0.653	0.297–1.436	0.289
Needed to visit doctor for physical health problem			0.005			0.009
No	Ref	Ref	–	Ref	Ref	–
Yes	2.202	1.270–3.818	0.005	2.250	1.225–4.132	0.009
Knowledge of where to access healthcare in Trinidad and Tobago upon arrival			0.792			0.814
No	Ref	Ref	–	Ref	Ref	–
Yes	1.119	0.485–2.578		0.892	0.344–2.312	0.814
Had difficulties accessing healthcare since in Trinidad and Tobago			0.260			0.037
No	Ref	Ref	–	Ref	Ref	–
Yes	1.399	0.780–2.508	0.260	0.463	0.225–0.954	0.037
Most trusted source of information on medical needs			0.014			0.790
Official healthcare providers	Ref	Ref	–	Ref	Ref	–
Social networks	0.437	0.170–1.126	0.086	0.525	0.147–1.882	0.323
Internet	0.373	0.193–0.719	0.003	0.862	0.404–1.836	0.699
Other	0.477	0.157–1.452	0.192	–	–	–

Migrants’ belief that they needed to visit a doctor for their physical health problems was significantly associated with their decision to seek public health services in Trinidad and Tobago (*p* = 0.005). The odds of migrants who believed that they needed to visit a doctor for their physical health problems seeking public health services were over twice the odds of migrants who did not have this perception (OR 2.202, 95% CI 1.270–3.818). The main source of information relating to medical needs was significantly associated with a migrant’s decision to seek public health services (*p* = 0.014). Migrants trusting the internet for information relating to their medical needs had lower odds of deciding to seek public health services relative to persons who trusted official healthcare providers (OR 0.373, 95% CI 0.193–0.719). Other variables relating to the health status and health needs of migrants were not found to be statistically significantly associated with their decision to seek public health services (*p* > 0.05).

Migrants’ belief that they needed to visit a doctor for their physical health problems was also found to be significantly associated with their decision to seek private health services (*p* = 0.009). The odds of migrants believing that they needed to visit a doctor for their physical health problems seeking private health services were over twice the odds of migrants who did not have this perception (OR 2.250, 95% CI 1.225–4.132). Having difficulties accessing healthcare since migrating to Trinidad and Tobago was another independent factor significantly associated with a migrant’s decision to seek private health services in the country (*p* = 0.037). Migrants who experienced difficulties accessing healthcare had lower odds of deciding to seek private health services (OR 0.463, 95% CI 0.225–0.954) relative to persons who did not have difficulties. A migrant’s self-rated health status, having diagnosed mental and physical health problems, needing to visit a doctor for their mental health problem and most trusted source of information for their medical needs were not found to be statistically significantly associated with their decision to seek private health services in Trinidad and Tobago (*p* > 0.05).

### 3.6. Andersen’s Behavioral Model for Health Service Use

Table 7 provided the results of our multivariable analyses of migrants’ decisions to seek public and private health services with variables selected from Andersen’s Behavioral Model for Health Service Use using backward elimination.

**Table 7:** Adjusted odds ratios (with 95% CI) of migrant’s decision to seek public and private health services using selected variables from Andersen’s Behavioral Model.

Variable	Decision to seek public healthcare (Ref: Decided not to seek public healthcare)			Decision to seek private healthcare (Ref: Decided not to seek private healthcare)		
	Adjusted OR	95% CI	*p*-value	Adjusted OR	95% CI	*p*-value
Time since migrating to Trinidad and Tobago			0.031	–	–	–
<12 months	Ref	Ref	–	–	–	–
≥12 months	2.336	1.083–5.039	0.031	–	–	–
Main reason for migration			0.489	–	–	–
Economical/Political	Ref	Ref	–	–	–	–
Asylum seeker/Escape violence	0.451	0.122–1.665	0.232	–	–	–
Family reunification	–	–	–	–	–	–
Household dynamic			0.049			0.020
Lives alone	Ref	Ref	–	Ref	Ref	–
Lives with family/friends	2.595	1.005–6.696	0.049	0.312	0.117–0.830	0.020
Visited doctor for physical health problem			0.010			0.012
No	Ref	Ref	–	Ref	Ref	–
Yes	2.170	1.203–3.913	0.010	2.263	1.199–4.272	0.012
Had difficulties accessing healthcare since in Trinidad and Tobago	–	–	–			0.045
No	–	–	–	Ref	Ref	–
Yes	–	–	–	0.466	0.221–0.983	0.045
Most trusted source of information on medical needs			0.037			0.714
Official healthcare providers	Ref	Ref	–	Ref	Ref	–
Social networks	0.450	0.162–1.252	0.126	0.472	0.125–1.785	0.268
Internet	0.409	0.205–0.816	0.011	0.809	0.365–1.794	0.602
Other	0.412	0.118–1.435	0.164	–	–	–

The predisposing, enabling and need factors of Anderson’s Behavioral Model based on the variables in our study were initially included in a multivariable binary logistic regression model to determine the odds of migrants deciding to seek public and private health services in Trinidad and Tobago. Backward elimination was employed to select the factors which best explained their decision to access these health services.

The predisposing factors relating to the length of time migrants resided in Trinidad and Tobago (*p* = 0.031), household dynamic (*p* = 0.049); the enabling factor relating to a migrant’s main source of information for medical needs (*p* = 0.037); and the need factor relating to visiting a doctor for physical health problems (*p* = 0.010) were observed to be significant correlates of a migrant’s decision to seek public health services in Trinidad and Tobago.

While adjusting for their reasons for migration, the odds of migrants living in Trinidad and Tobago for more than 12 months seeking public health services remained over twice the odds of migrants residing in the country for less than 12 months (AOR 2.336, 95% CI 1.083–5.039). The odds of persons living with family or friends seeking public health services were now over two times the odds of persons living alone (AOR 2.595, 95% CI 1.005–6.696). The odds of migrants who believed that they needed to visit a doctor for their physical health problems seeking public health services remained over twice the odds of migrants who did not have this belief (AOR 2.170, 95% CI 1.203–3.913). Migrants who trusted the internet for information on their medical needs seeking public health services continued to have lower odds of deciding to seek public health services relative to persons who trusted official healthcare providers (AOR 0.409, 95% CI 0.205–0.816).

The predisposing factor of a migrant’s household dynamic (*p* = 0.020) and the enabling factor of having difficulties accessing healthcare (*p* = 0.045) were observed to be significant correlates of a migrant’s decision to seek private health services in Trinidad and Tobago.

While adjusting for their sources of information for medical needs, the odds of migrants living with family or friends and seeking private health services remained lower than the odds of migrants living alone (AOR 0.312, 95% CI 0.117–0.830). Similarly, the odds of migrants who experienced difficulties accessing healthcare while in Trinidad and Tobago then deciding to seek private health services remained lower than the odds of persons who did not have difficulties (AOR 0.466, 95% CI 0.221–0.983).

## 4. Discussion

It has been emphasized that migrants face multiple barriers to care and health services throughout their migration journey and in their country of destination (9, 10). Using constructs of Andersen’s Behavioral Model for Health Service Use, this study aimed to examine the medical needs and associations between predisposing, enabling and need factors on decisions to seek public and private health services among a sample of Venezuelan migrants residing in Trinidad and Tobago.

With the inflows of Venezuelan refugees and migrants to Trinidad and Tobago, there has been increasing demands on the use of health services in the public sector. The health sector of Trinidad and Tobago is comprised of public and private care systems and all nationals may access care through the public system at no cost. At the primary level, healthcare is offered through district health centers, with some providing 24-h access to services. Additional primary care and treatment services for selected conditions are offered through non-governmental organizations which are supported by the Government. Secondary care services are provided by public hospitals and through inpatient and outpatient clinical services. General hospitals are often the first point of offering immediate/emergency care for physical injuries, inpatient and outpatient medical and surgical services, psychiatric inpatient and outpatient services and linkages to specialist referrals. Specialist hospitals offer a range of services including psychiatric, women’s and maternity care. A selected range of tertiary care services is also offered at specific hospitals. Some tertiary level services such as cardiac surgery and magnetic resonance imaging (MRI) scans have been offered to the public. The private health sector is smaller and includes a variety of healthcare providers such as physicians, dentists, pharmacists, opticians, along with private healthcare facilities including private hospitals and nursing homes, clinical laboratories, and diagnostic testing facilities. Most tertiary care services are accessed privately or abroad and paid for either out of pocket or through private insurance.

The Government of the Republic of Trinidad and Tobago developed a draft policy for treating with non-nationals with respect to providing public healthcare services. The policy indicated that registered Venezuelan migrants were to be granted access to free emergency medical services, primary healthcare, and immunization services available in the public sector. Apart from this, Venezuelan migrants and non-nationals benefitted from the Government’s “Treat All Policy” with respect to non-communicable diseases. Given increasing constraints on the public health system, the policy further advised that Venezuelan migrants, refugees, and other non-nationals seeking ongoing medical care and/or specialized health services may do so at their own costs. In this regard, migrants and refugees would resort to paying for private health care services to address their unmet medical needs.

Participation in our study was not linked to the Government Registration Exercises and, therefore, the study did not ascertain how many migrants in our sample were part of these undertakings. Overall, over two thirds of migrants in our study reported that they used public health services, while around one-quarter indicated they used private health services. As it pertained to their medical needs, 30% of migrants reported being worried about diagnosed mental and physical health problems. Additionally, 21.2% of the migrants reported needing to visit a doctor for a mental health problem and 45.2% for a physical health problem since residing in Trinidad and Tobago. When asked to rate their health, 10% of participants rated themselves as “not healthy,” with 30% rating themselves as being in “fair health.” Our findings suggested that almost half of our study population reported experiencing health challenges which required medical attention. This may be reflective of their unmet health needs. Many refugees and migrants with medical conditions experienced interruptions in access to healthcare because of their migration journey. Refugees and migrants may also experience new health problems because of the social and economic hardships experienced in the host countries, leaving them potentially at a greater risk for contracting infectious diseases due to their exposure to infections and lack of health resources (9–11).

The results of our univariate analyses showed significant associations between a migrant’s decision to seek public health services with the length of time residing in Trinidad and Tobago, living with family/friends, monthly earnings, visiting a doctor for a physical health problem and their most trusted source of information on their medical needs. A migrant’s decision to seek private health services was significantly associated with living with family and friends, visiting a doctor for a physical health problem and having difficulties accessing healthcare in Trinidad and Tobago.

In the context of Trinidad and Tobago, recently arrived migrants received information on services available to them at the time of pre-registration by the UNHCR and local implementing partners. These services included legal assistance, cash-based assistance, refugee status determination services, case management, psychosocial support, linkage to assistance for medical services including survivors of gender-based violence (GBV), sexual and reproductive health services, employment skills training and English language training. These services also covered information about the availability of medical services offered in the public system and through UNHCR supported NGO networks. Persons arriving with and living with families were provided with information on the availability of healthcare services in the public health system. In addition, just over half of the migrants enrolled in our study reported having an income of $1,000 TT or less which may explain the association between monthly earnings and their decisions to access healthcare in the public system versus the private system. Having visited a medical doctor in the past 12 months suggested an indication of their medical needs which was further associated with the decisions to use of both public and private health care systems.

Our study results also highlighted the importance of social networks in decisions to use private health services by migrants, further underscoring the value of family and friends in helping to steer individuals to seek healthcare. The use of the private healthcare services was also associated with migrants who experienced difficulties accessing healthcare services in general, suggesting challenges related to accessing the health system due to barriers based on registration status and/or medical needs due to the limitations imposed by the Government Policy which did not facilitate the provision of ongoing medical and/or specialized care to Venezuelan refugees and migrants and other non-nationals in the public setting.

These findings were further supported in studies which presented that migrants and refugees’ decisions to seek healthcare could be compromised because of social characteristics when interacting with the health system in host countries. Factors such as employment, level of earnings, educational background, language barriers and information on where to access health services upon arrival in countries have impacted migrants’ use of health services (12, 13).

Andersen’s Behavioral Model and its expanded versions have been widely used across various health service settings and across several populations both in clinical and non-clinical settings to understand the factors associated with health service use. The findings of existing studies showed mixed results, each underscoring the relative contributions and significance of the model’s theoretical constructs, i.e., predisposing, enabling and need factors, in explaining disparities in the uptake and use of mental health services, antenatal care, long-term care and the overall health seeking behaviors among various populations (14–23). In our study, the theoretical constructs of Andersen’s Behavioral Model namely the predisposing factors of length of time residing in Trinidad and Tobago and living with family/friends; the enabling factor of receiving information from public healthcare providers; and the need factor of visiting a doctor for a physical health problem were significant correlates of migrants’ decisions to seek public health services. This study also found that predisposing factors such as living with family/friends and the enabling factor of having difficulty accessing healthcare services were significant determinants of their decision to seek services from private health care providers.

Our findings reinforced literature which employed Andersen’s theoretical constructs to examine the health seeking behaviors among migrant subpopulation groups. Studies supported that the model’s constructs namely predisposing factors and need factors were consistently and significantly associated with migrants’ use of health services. One study using a cross-sectional sample among migrant workers in Malaysia examined the influence of sociodemographic factors on the use of health services. Guided by Andersen’s Behavioral Model, the results showed that predisposing factors namely marital status and education, and need factors namely self-rated health status, sickness, and chronic illnesses in the previous year were significant determinants of health service use among the study participants (20). In another study based on a stratified sample of migrants in Beijing, Andersen’s Behavioral Model was also used to demonstrate the effects of migrants’ predisposing, enabling and need characteristics on their use of health services. The results of their binary logistic regression showed that the predisposing variable of ethnicity and need variable defined by “degree of symptom” were significant correlates of health service use and consistently predicted the uptake of health services among migrants (22). In our application of Andersen’s Model, the predisposing factor of living with family/friends and the enabling factor of having difficulty accessing health services were significant correlates influencing migrants’ decisions to seek private health services. The results underscored the importance of migrants’ social networks, i.e., family and friends, in the designing and planning of interventions to increase service uptake and reducing barriers to health service use. Therefore, to increase access to and the uptake of health services, it may be helpful to reach migrants through targeted communication campaigns with their social networks and wider community.

Other studies have highlighted the importance of need factors in predicting migrants’ use of health services. In one study among African immigrants living in the United States, Andersen’s Behavioral Model was used to explore factors associated with the use of and barriers to the uptake of mental health services. The results of their analysis showed that need and enabling factors such as age, religion, acculturative stress and neighborhood risk factors were most common in predicting mental health service use, while hope of self-healing and financial factors were reported as the most common barriers to the uptake of mental health services (23). In a more recent study, Andersen’s Behavioral Model was also used to explain the utilization of health services and examine gender and ethnic differences in service uptake among Middle Eastern, Hispanic/Latino and Asian immigrants in the United States. Overall, the study found that Andersen’s constructs namely the need factor of the likelihood of seeing a doctor in the past 12 months significantly predicted health service use among Middle Eastern immigrants. All immigrant women, regardless of ethnicity, were more likely than men to report seeking medical services and the effect of predisposing, need and enabling characteristics for Hispanic and Asian immigrants were significantly different to Middle Eastern immigrants (15). Therefore, our findings corroborated with the existing literature that predisposing, enabling and need factors were vital in examining migrants’ decisions to use health services in host countries.

The influence of these social characteristics, resource availability and quests for solutions to illnesses on the uptake of health services by migrants has been supported in studies of undocumented migrants as well (24, 25). Our study showed that there was a greater tendency for migrants to use public health services, i.e., hospital and emergency rooms when they had a physical health problem that required medical attention. This finding also suggested that migrants were treating physical non-communicable health problems more seriously (by visiting a doctor). Studies have emphasized the increased need for culturally appropriate health services for refugees and migrants assisting to reduce the risk of both mental and physical illness and increase service uptake by those who needed it (26, 27).

Our study was conducted in 2020, i.e., one year after the Government Registration Exercise granting access to free primary health care and emergency medical services for Venezuelan migrants and families who were successfully registered. This may help explain why migrants living with family or friends displayed higher odds of seeking public health care relative to those living alone. It is possible that family and friends benefitted from the Government Registration exercise granting access to free publicly available health care. Alternatively, our results also showed that the odds of using private health care were lower among migrants living with family and friends than persons living alone suggesting that the use private healthcare was a deterrent for migrants living with other persons given the associated costs.

To the best of our knowledge, this was the first study aimed at examining the decision to seek public and private health services by the Venezuelan migrant subpopulation in the English-speaking Caribbean. Our study provided vital insights into the health needs of these migrants while applying Andersen’s Behavioral Model to this vulnerable subpopulation. However, further studies are needed to understand specific barriers influencing migrants’ decisions to seek health services, identify their needs for ongoing care and to inform evidence-based targeted interventions and programs addressing their health needs.

### 4.1. Study limitations

One of the limitations of this study was the sample size of *n* = 250 participants. There are thousands of undocumented migrants from Venezuela in Trinidad and Tobago as in the case of the Government Registration Exercise in which over 16,000 migrants participated. Therefore, the sample is not generalizable to the larger population of Venezuelans living in Trinidad and Tobago. Another, limitation was that some of these findings may be biased in part due to the selection of study participants. Some the migrants elicited for this study were selected from persons who previously accessed outreach health services and who were seeking refugee status documentation.

However, despite these limitations our study was vital as it examined the medical needs and factors associated with the uptake of health services among a highly vulnerable subpopulation. This has not been previously explored in Trinidad and Tobago and the wider English-speaking Caribbean. All migrants who were approached participated in the study and we are confident that it yielded value as migrants are regarded as a hidden population given levels of stigma and many of whom did not want to access public health services because of their legal status. Our study did not consider whether migrants had health insurance, nor did we assess the role of stigma, but these migrants may have also experienced difficulties while using public health services due to societal and/or internalized stigma.

## 5. Conclusion

The findings from our study were comparable to results of the IOM Displacement Tracking Matrix Survey showing that of the migrants who accessed health services, 53% reported going to public hospitals and 39% reported going to public health centers (28). A total of 1,323 Venezuelan nationals living in Trinidad and Tobago were surveyed between November and December 2022. The available data showed a large proportion of Venezuelans using publicly available health services and by extension suggested an increasing demand on the public health sector to provide these services. Given the high demand for the use of public healthcare services, our study aided in understanding the scope of their medical needs and the factors associated with their decision to use these public and private sources of healthcare services.

Overall, our findings contributed to the existing body of literature underscoring the value of Andersen’s Behavioral Model for Health Service Use. In addition to migrants’ predisposing, need and enabling characteristics, factors related to the macro environment such as Government Policies, may additionally account for variations in health service use (29). In the context of our study, Venezuelan migrants benefitted from Government Policies which facilitated their access to primary care and emergency health services in the public sector and relied on public information about the availability of health services for their medical needs. Additionally, during the time of public health emergencies such as in the case of the COVID-19 pandemic, refugees and migrants faced worsening health conditions and increasing risks because of their access to and availability of public healthcare services. Therefore, without adequate policies, migrants and refugees find themselves in a “tolerated” situation with limited access and information on the availability of health care services leaving them with fewer resources to maintain their health and reduce the burden of non-communicable diseases.

## Data availability statement

The data analyzed in this study is subject to the following licenses/restrictions: The raw data supporting the conclusions of this article will be made available by the authors, without undue reservation. Requests to access these datasets should be directed to b.bhagwandeen@hw.ac.uk.

## Ethics statement

The studies involving humans were approved by University of the West Indies (UWI) Institutional Review Board (IRB). The studies were conducted in accordance with the local legislation and institutional requirements. The participants provided their written informed consent to participate in this study.

## Author contributions

NL conceived and designed the study, collected, and discussed the data, wrote, discussed, and reviewed the manuscript. BB conducted the analysis and interpreted data, discussed, wrote, and reviewed the manuscript. All authors contributed to the article and approved the submitted version.
